# Dyslexic learners’ experiences with their peers and teachers in special and mainstream primary schools in North-West Province

**DOI:** 10.4102/ajod.v7i0.363

**Published:** 2018-03-05

**Authors:** Monicca Leseyane, Peter Mandende, Mary Makgato, Madoda Cekiso

**Affiliations:** 1 Department of Applied Languages, Tshwane University of Technology, South Africa

## Abstract

**Background:**

Inclusive education requires that the framework within which education is delivered should be broad enough to accommodate equally the needs and circumstances of every learner in the society. This includes learners with disabilities like dyslexia who have been excluded from the formal education system. This article reports the findings of a qualitative study that explored and described the dyslexic learners’ experiences with their peers and teachers in special and public schools in North-West Province of South Africa.

**Methods:**

The study adopted a qualitative methodology and used a phenomenology research design. The sample was purposively selected and comprised nine dyslexic learners. All the learners were in public schools previously and were later moved to a special school after being diagnosed as dyslexic. The participants were aged 9–12 years. The researchers conducted one-on-one interviews with the participants and content-analysed the data.

**Findings:**

The findings revealed that in public schools the dyslexic learners were exposed to ill-treatment by other learners who despised, ridiculed, bullied and undermined them. The findings further revealed that teachers in public schools were not patient with dyslexic learners, did not give them extra attention and that some teachers used negative comments that embarrassed them.

**Conclusion:**

The article spells out the barriers experienced by dyslexic learners in public schools and also recommends training of teachers so that they know how to deal with dyslexic learners, thereby eliminating the barriers. The study further recommended awareness campaigns among the student body about dyslexia.

## Introduction

South Africa followed international trends in accordance with the social rights discourse and adopted inclusive education. Subsequently, the policy document, Education White Paper No. 6 (2001), was developed, and it outlined and accepted its responsibility to provide a supportive inclusive education environment for learners with special needs (Sukhraj 2006). In this context, inclusion is broadly understood as the process by which learners who previously might have been taught in a separate special education system because of the barriers to learning they experience would now be taught in regular schools (Walton et al. 2009). Similarly, Olagboyega (2008) believes that inclusive learning states that learners with learning difficulties, such as dyslexia, do not necessarily require specialist additional support in order to gain access to the curriculum but the process of teaching and learning needs to be broadened so that such learners can be included within it. However, Prinsloo (2001) and Peters (2007) are of the view that despite the introduction of inclusive education policy in South Africa, it is clear that a number of groups remain vulnerable. These include children with disability and those children who for a variety of reasons experience barriers to learning. This idea is echoed by Selvan (2004) in Mweli and Kalenga (2009) who observes that the majority of learners who experience learning difficulties or are physically disabled have negative experiences within the school environment. Selvan further states that these learners are being laughed at by their peers and are labelled and excluded in peer-group tasks and activities assigned in the classroom. Moreover, Bhengu (2006) found that children with disabilities are not easily accepted in regular classes.

It is against this background that the current study seeks to explore and describe the dyslexic learners’ experiences with their peers and teachers in special and public schools. Specifically, this study explores the classroom experiences of dyslexic learners with a view to establishing if the classroom environment in public schools is conducive for them to learn as stated in the inclusive education policy. It is assumed that having this knowledge would assist the inclusive education implementation strategy by identifying the needs of the dyslexic learners and therefore devise means to address them. In addition, Hoskins (2015) points out that the results of research on dyslexic learners is likely to equip professionals such as psychologists, medical practitioners and therapists, researchers and adults who have dyslexic children. The vast amount of research on the implementation of inclusive education has focused on teacher preparation to teach the learners with disabilities (Sukhraj 2008; Walton et al. 2009) and the suitability of the schools’ infrastructure for such learners. Hoskins (2015) believes that the bulk of research focuses on identification, causes and assessment (Burden 2000), while others focus on the effects that dyslexia has on self-esteem (Alexander-Passe 2006; Gibson & Kendell 2010; Glazzard 2010). However, Davie in Burden et al. (2005) is of the view that a neglected source of valuable information regarding the effectiveness of educational policies and interventions needed for learners with special needs has been the voices of these learners themselves. Justifying the importance of the learners’ voice, Burden et al. (2005) state that the importance of the voice of the learners being used has been indicated by many researchers, claiming that school experiences need to be told by the learners while still in school so that their emotions, challenges and needs are understood. In addition, Glazzard (in Hoskins 2015) points out that the voice of the learners with dyslexia can be used to discover the ways in which schools and teachers can more effectively meet their needs. Few studies have focused on the classroom experiences of the dyslexic learners with their peers. Among the few studies is the study conducted by Hoskins (2015) on the experiences of Grade 6–9 dyslexic school learners in South Africa.

### Definition of dyslexia

There are numerous definitions of dyslexia by different authors. Dyslexia is a specific learning disability that is neurobiological in origin. It is characterised by difficulties with accurate or fluent word recognition and by poor spelling and decoding abilities. These difficulties typically result from a deficit in the phonological component of language that is often unexpected in relation to other cognitive abilities and the provision of effective classroom instruction (Lyon et al. 2003). In addition, Stanovich and Siegel in O’Brien, Mansfield and Legge (2005) state that a phonological processing deficit impedes a child’s ability to develop graphene-phoneme correspondence rules, and to decode words. The International Dyslexia Association (n.d.) is of the view that prior research has focused more on the reading than the spelling problems of learners with dyslexia. They point out that written spelling also poses problems to learners with dyslexia. Olagboyega (2008) challenges the definitions that view dyslexia as just a problem and suggests that dyslexia be defined in terms of differences in cognition and learning rather than deficits. Subsequently, Olagboyega defines dyslexia as a complex neurological condition which is constitutional in origin and may affect oral language skills, motor function, organisational skills and numeracy.

### Symptoms of dyslexia

Moats et al. (2010) identify the symptoms of dyslexia. They state that the primary symptoms of dyslexia are inaccurate or slow printed word recognition and poor spelling problems that in turn affect reading fluency, comprehension and written expression. They further point out that other types of reading disabilities include specific difficulties with reading comprehension or speed of processing (reading fluency). In contrast, Olagboyega (2008) believes that the characteristics of dyslexia may include a discrepancy between ability and standard of work produced, a discrepancy between intelligence and ability to learn, a problem with memory and word retrieval, a problem with speed of reading and processing meaning often because of an inability to break down words morphologically. He also mentions the difficulties with spelling even of an easy word as one of the characteristics of dyslexia. He further points out that such spelling difficulties include misrepresentation of the sound, for example, ‘pad’ for ‘pat’; wrong word boundaries, for example, ‘firstones’ for ‘first ones’; wrong syllabification, for example, ‘rember’ for ‘remember’; wrong doubling of letters, for example, ‘eeg’ for ‘egg’; intrusive vowels, for example, ‘tewenty’ for ‘twenty’; ‘b’, ‘d’ confusion, for example, ‘bady’ for ‘baby’; and letter reversal, for example, ‘lentgh’ for ‘length’ or ‘tow’ for ‘two’. In addition, Lynn (2000) is of the view that dyslexic learners sometimes feel very different from their peers simply because they may be unable to follow simple instruction, which for others seems easy.

### The impact of dyslexia on reading and writing

The ability to read and write is recognised as being one of the most fundamental of the core skills contributing to academic achievement, lifelong learning and sustainable development (Trudell et al. 2012). However, learners with dyslexia find it difficult to achieve or obtain this goal as every subject requires some reading and writing. Specifically, the Regents of the University of Michigan (2016), in their recent research, are of the view that dyslexia makes it difficult for learners to master the following skills which are crucial to the learning process: access to written texts, reading fluency, spelling, organising information, following written directions and sequencing information. They further point out that as a consequence of their reading difficulties, learners with dyslexia are forced to compensate for their weaknesses by following their peers, verbally processing information, relying on rote memorisation and using hands-on or experiential learning contexts. Accounting for the problems of dyslexia that are related to the learning process, Asiko (n.d.) who is the chief executive of the non-profit Strive International that seeks to improve the educational experience of learners with dyslexia in Africa claims that in South Africa 1 in 10 people are dyslexic. Thus, approximately 5 million South Africans are struggling with literacy problems in school or at the workplace. Accounting on the problems of dyslexia to learning, Lynn (2000) claims that with an ever increasing emphasis on education and literacy, more and more children and adults need help in learning to read, spell, express their thoughts on paper and acquire use of grammar. She further states that dyslexic children who find the acquisition of these literary skills difficult can also suffer much anguish and trauma when they may feel mentally abused by their peers within the school environment, because they have a learning difficulty.

Dyslexia affects the reading process at two levels, that is, decoding and reading comprehension (Gough & Turner in Pirttimaa, Takala & Ladonlahti 2015). In addition, Pirttimaa et al. (2015) argue that dyslexia is mainly caused by problems in phonological coding and the persistence of poor phonological skills. They further point out that problems with phonological decoding lead to difficulties in connecting spoken and written words. This idea is supported by Elbro and Scarborough (2004) who state that problems with phonological decoding and other challenges in phonological ability seem to be the core deficit in dyslexia. Ransby and Swanson in Pirttimaa et al. claim that better reading achievement is associated with better phonological awareness and more fluent rapid naming. They further point out that problems with reading comprehension seem to include persistent deficits not only in word recognition skills but also in vocabulary, working memory and listening comprehension. The Access Ability Centre (n.d.) believes that learners with dyslexia also experience problems with composition. They believe that this problem may be accompanied by difficulty with spelling and handwriting and as a result learners may choose words they can spell rather than those they want to use. The Access Ability Centre further observed that learners with short-term memory problems may have difficulty transcribing a mentally composed sentence, thus much backtracking is required which disrupts the flow of thought. In a study conducted by Hudson, High and Otaiba (2007), they observed that children with dyslexia often show two obvious difficulties when asked to read text at their grade level. First, they will not be able to read as many of the words in a text by sight as average readers. They further state that there are always many words on which the dyslexic learners stumble, guess at or attempt to sound out. Second, Hudson et al. observed that writing letters and words backwards are common in the early stages of learning to read and write among average dyslexic learners.

### Voices of dyslexic learners

Davie in Hoskins (2015) believes that a neglected source of value information regarding the effectiveness of educational policies and interventions needed for learners with special needs has been the voices of these learners themselves. This view is supported by many researchers like Bearne (2002), Casserly (2011) and Glazzard (2010). According to Burden (2000) in Hoskins (2015), the bulk of research focuses on identification, while other researchers focus on the effects that dyslexia has on self-esteem. However, Gibson and Kendall (2010) in Hoskins (2015) are of the view that despite all the research that has already been carried out with dyslectic learners, literature using the voice of learners about their school experiences is scant and limited, hence the need for the current study. The scantiness of information about the experiences of the dyslexic learners is further supported by Nugent (2008) and Humphrey (2003). In addition, Gunnel Ingesson in Hoskins (2015) believes that research on learners with dyslexia is frequently based on parent–teacher ratings and rarely based on accounts of the affected persons themselves. Therefore, the current study seeks to answer the following research questions:

- How do dyslexic learners relate with their peers and teachers in public schools?
- How do dyslexic learners relate with their peers and teachers in special schools?
- Which learning environment do dyslexic learners believe to be conducive between public and special schools?

### Problem statement

Murungi (2015) points out that the Salamanca Statement and Framework for Action proclaimed that the regular schools with inclusive orientation are the most effective means of combating discriminatory attitudes, creating welcoming communities, building on inclusive society and achieving education for all. However, the results of the study conducted by Nugent (2008) reveal that learners in special schools and reading units seem to be happier and have more positive experiences than those in mainstream schools. This view is reiterated by Riddick (2010) who observed that learners with special needs attending mainstream schools are more likely to experience bullying from their peers. In the current study, the focus is on the experiences of dyslexic learners about both their peers and teachers in special and mainstream schools.

### Rationale of the study

Research has shown that dyslexic learners require a classroom environment which is predominantly a learning environment where they can feel comfortable and develop confidence and self-esteem (Lynn 2000). Therefore, such an important resource as learning environment should be left to the dyslexic learners to identify. Subsequently, the current study sought to take the dyslexic learners’ voices into consideration in an attempt to understand and prescribe a learning environment for the dyslexic learners. Having this valuable information is likely to assist policymakers in designing appropriate and informed policies, and the department of education could design appropriate interventions and also empower teachers on how to handle dyslexic learners in their classrooms.

## Research method

### Design

The study was qualitative in nature, and it followed a phenomenological research design. Cohen, Manion and Morrison (2007) define phenomenology as a theoretical point of view that advocates the study of direct experience taken at face value and one which sees behaviour as determined by the phenomena of experience, rather than by an external, objective and physically described reality. In addition, Groenewold (2004) states that phenomenology aims to accurately describe the phenomenon, remain true to the evidence and understand it from the perspectives of the people involved. This research design was felt relevant for the current study as it allowed the researchers to focus on the subjectivity of the viewpoints and experiences of the dyslexic learners as they were viewed as being the best authorities of their lives.

### Subjects

The sample consisted of nine dyslexic learners. These learners were purposefully selected as they were formally diagnosed with dyslexia by psychologists in their previous schools before joining the special school and as such all the respondents were aware of their condition. The learners were enrolled for grades 3 and 4 at a special school together with learners with other challenges, including visually impaired learners and physically challenged learners who had learning challenges. The sample consisted of three girls and six boys and their ages ranged from 9 to 12 years. The mother tongue of all the respondents was Setswana.

### Instrumentation

Semi-structured interviews were used in order to get information from the respondents. According to Hall (2017), semi-structured interviews use an interview guide with some questions developed in advance and also allow the interviewer to stray from the interview guide, asking follow-ups as the interviewer believes appropriate. This type of interview was chosen because it is an easy, reliable method; both persons can see one another and if the respondent finds it hard to understand the question, time and space allow the researcher to rephrase the question without both of them being under pressure.

### Data analysis

The recorded interviews were transcribed and translated into English. One of the researchers who spoke Setswana translated the interviews back into Setswana in order to eliminate inconsistencies. After the researchers had satisfied themselves that the transcript was accurate, they made four copies of it and independently coded the data. After coding the data, the researchers held a meeting where they discussed the themes and reached a consensus on five themes.

## Ethical considerations

The clearance certificate to conduct this study was granted by the Tshwane University of Technology’s (TUT) Faculty Committee for Research Ethics-Humanities (FCRE-HUM). This committee is a subcommittee of the Senate Committee for Research Ethics. The TUT Research Ethics Committee is registered with the National Health Research Ethics Council (REC-160509-21). The clearance certificate was submitted to the school principal who later granted permission to one of the researchers to conduct research at the identified school. As already mentioned, the respondents were aged between 9 and 12 years, which meant that the researchers had to seek permission from the parents or guardians. In the consent letter, the purpose of the study and the rights of the respondents were explained. After the parents or guardians had allowed learners to participate in the study, one of the researchers whose mother tongue is Setswana scheduled an appointment with the school principal. The dyslexic learners were identified with the assistance of the educational psychologist based at the special school. The researcher interviewed each respondent for about 30 min in their mother tongue. The researcher explained the purpose of the study and sought permission from the respondents to record the interview, which was granted by all interviewees. All respondents were interviewed in a day.

## Findings

### Relationship between dyslexic learners and their peers in public schools

The dyslexic learners painted a gloomy picture about the relationship with their peers in public schools. They indicated that many learners in public schools did not understand their problem as dyslexic learners. As a result, when they battle to read and write, they become the centre of attraction among the normal learners. The dyslexic learners mentioned that very few learners in the public school sympathised with them, but the majority teased and laughed at them. This attitude made them look inferior to other learners. The majority of the dyslexic learners felt embarrassed by the fact that they were different from other learners in terms of reading and writing performance. Being different made them uncomfortable and they lost confidence in themselves. This negative attitude was facilitated by the negative comments they received from their peers emanating from the fact that they struggled a lot with reading and writing. They further mentioned that when they were grouped by their teachers in class they felt embarrassed as their peers sometimes would like them to give feedback on behalf of the group. In such situations, they felt that their failure to read and write properly did not only affect them but also their peers. They also mentioned that in public schools they were bullied by other learners as they looked inferior as far as their academic performance was concerned. One respondent said:

‘When I was at public school my peers used to make me feel stupid. When I struggle to read or mispronounce words they would laugh at me. This worried me a lot as I was the only one in class experiencing this problem. As a result I decided to isolate myself so as to avoid embarrassment. Even at home I used to isolate myself from playing with other children since I had developed a very low self-esteem.’ (Participant 4, male, grade 3)

On the same question, another respondent mentioned the following:

‘Other learners used to judge me because they did not understand my problem. They made me feel like blaming myself and yet I did not choose for myself to have these challenges. The situation was worse when it came to group work. My group members would force me to give feedback representing our group. This was a strategy to attract everybody’s attention to my challenges as the whole class would laugh at me.’ (Participant 1, female, grade 4)

Another respondent said:

‘Observing other learners reading properly was frustrating. This made me feel less than other learners in class. My major problem was with spelling, especially English words. My problem was better in the mother tongue.’ (Participant 9, female, grade 3)

### Relationship between dyslexic learners and educators in public schools

The dyslexic learners complained about the manner in which they were treated by the majority of teachers in public schools. They mentioned that teachers were not patient with them. They felt that their teachers did not give them extra attention but treated them like other learners in the classroom. They felt that teachers in the public school did not understand that they have learning challenges and were different from other learners and therefore needed special attention. They further mentioned that some teachers used negative comments that embarrassed the dyslexic learners. One learner said:

‘In my previous school some teachers used to be angry and punish me when I failed to read and write properly. They seemed not to understand that I had a challenge. They thought that I was stupid in class and I was there to create problems for them. They also felt that I was holding back the class as they were no longer moving at their normal pace in an attempt to accommodate me.’ (Participant 2, male, grade 4)

On the same question another learner said:

‘Teachers used to ridicule me in front of other learners. They would ask me to read alone while other learners were listening and this embarrassed me. What frustrated me most was to fail to imitate the teacher when she was modelling reading for me. As she was paying attention to me some learners would feel bored and tease me during break.’ (Participant 8, female, grade 3)

Responding to the same question, one learner said:

‘I did not like the manner in which I was treated by the majority of teachers. They made me feel inferior and stupid. I did not receive any support from them. As a result I used to bunk school because I was not happy at all. At some stage I thought of dropping out but my parents promised to take me to a special school.’ (Participant 6, male, grade 3)

### Relationship between dyslexic learners and their peers in special schools

Dyslexic learners expressed satisfaction about their relationship with their peers in special schools. They emphasised that the very fact that there were many children like them in such schools made them feel comfortable. In special schools, they did not see themselves different from other learners as used to be the case in public schools. Other students seemed to understand their challenge and did not ridicule them. Instead, they received support from their peers. The fact that they were not different from other learners in their classroom made them feel normal. One learner said:

‘I have been here for 6 months and I am comfortable because my peers recognise me as a human being. That makes me feel comfortable and I have regained the confidence I lost when I was in a public school. Other learners do not laugh at me here.’ (Participant 8, female, grade 3)

Responding to the same question, another learner said:

‘Other students are patient with me. They seem to understand what I am going through and the very fact that I am not the only one with this challenge makes me feel comfortable. There are many of us here who battle with reading and writing and that makes me feel not different.’ (Participant 6, male, grade 3)

On the same question, another learner said:

‘I am happy with the treatment and respect I receive from other learners here. They do not ridicule nor bully me as it used to be the case in public schools.’ (Participant 2, male, grade 4)

### Relationship between dyslexic learners and educators in special schools

The respondents expressed satisfaction with the manner they were treated by their teachers in a special school. Dyslexic learners felt that teachers in special schools understood their challenge and as such were patient with them. They also observed that teachers at a special school knew how to deal with their challenge as compared to teachers in public schools. Responding to the question, one learner said the following:

‘Teachers treat us well here. Since I came to a special school I am feeling well and my academic performance has picked up because sometimes I receive extra lessons.’ (Participant 3, male, grade 4)

On the same question, another learner said:

‘In the current school educators treat me normal like other learners but in the previous school I did not receive good treatment and that was the reason why I ended up losing interest in school. Another important thing is that teachers at special school do not see dyslexia as a challenge, they know how to deal with dyslexic learners. They make me feel welcome as I spend more hours with them than at home.’ (Participant 7, male, grade 4)

#### Learning environment that dyslexic learners prefer

The dyslexic learners preferred a special school environment to that of a public school. They explained that the public school environment was not friendly to them and did not allow them to prosper in their academic endeavours. They pointed out that the public school environment made them feel different from other learners, whereas the special school environment made them feel like normal human beings. They mentioned that in special schools they had the opportunity to interact with other dyslexic learners, something that did not exist in public schools. One learner said:

‘Based on my experience with a public school, I feel that the special school provides a better learning environment because it recognises me as a human being who has potential to learn. Moreover, the special school environment helps me develop a positive self-concept whereas the public school made me feel inferior to other human beings.’ (Participant 5, male, grade 3)

Responding to the same question, another learner said:

‘I think special school environment provides a conducive learning environment than public learning environment. I am saying this because my academic performance improved while attending a special school. Here, I feel confident and optimistic that I will be able to achieve the academic goals I have set for myself.’ (Participant 7, male, grade 4)

## Discussion of findings

The results of the study revealed that the relationship between the dyslexic learners and their peers was negative in the public school. These learners were exposed to ill-treatment by other learners who despised, ridiculed, bullied and undermined them. When dyslectic learners failed to read and write properly, they became objects of ridicule by their classmates who could read and write better than them. This finding is supported by Selvan (2004) in Mweli and Kalenga (2009) who observed that the majority of learners who experience learning difficulties or are physically disabled have negative experiences within the school environment. Selvan in his study further observed that these learners were being laughed at by their peers and were labelled and excluded in peer-group tasks and activities assigned in the classroom. This finding is echoed by Bhengu (2006) who found that children with disabilities were not easily accepted in regular classes. The finding on the negative relationship between dyslexic learners and their peers is further confirmed by Nugent (2008) who observed that dyslexic learners were exposed to distress, failure and in many cases bullying.

The results further revealed that because of the suffocating situation the dyslexic learners went through in public schools, they developed a negative self-concept. This finding is echoed by Riddick (2010) who argued that as dyslexia affects self-esteem, learners with reading and writing difficulties may develop social and emotional problems, including psychiatric problems. This finding is also supported by Burden (2000) who conducted a study with 50 dyslexic boys whose self-efficacy increased while attending a special school for children with dyslexia in comparison to the regular school they had previously attended.

It is also noteworthy that dyslexic learners were not satisfied with the manner in which they were handled by teachers in public schools. Specifically, they complained that teachers in public schools were not patient with them, did not give them extra attention and that some teachers used negative comments that embarrassed them. This finding is supported by Thompson (2013) who claimed that the level of teacher awareness of special needs education and in particular dyslexia needs to be developed.

The findings of the study further point to a positive relationship between dyslexic learners and their peers in a special school. This is partly owing to the fact that in special schools, the dyslexic learners interact with learners who are also dyslexic and therefore do not see themselves as different. This finding is echoed by Nugent (2008) who observed that the relationship between dyslexic learners and their peers who attended public school was negative although those who were in a special school setting were likely to have positive experiences with friends and developed a feeling that they were all in it together.

The results further revealed a positive relationship between dyslexic learners and their teachers in a special school setting. The dyslexic learners felt that teachers in a special school understood their challenges and as such were patient with them. They also observed that teachers at a special school knew how to deal with their challenges as compared to teachers in public schools. Olagboyega (2008) is of the view that a general awareness of the dyslexic continuum of characteristics is essential to the teacher as these characteristics may include a discrepancy between ability and standard of work produced and discrepancy between intelligence and ability.

The findings of this study further revealed that the dyslexic learners preferred a special school environment to that of a public school. They associated the special school environment with academic success. This finding is supported by Lynn (2000) who argued that in a positive and encouraging environment, a dyslexic child will experience the feeling of success and self-value. This finding is further supported by Nugent (2008) who observed that learners in special schools and reading units seemed to be happier and had more positive experiences than those in mainstream schools. A similar finding was observed by Hellendoom and Ruijssenaars (2000:233) in Hoskins (2015) who noted that one learner said the special school gave him a sense of himself and some sense of who he could be.

## Conclusion

Despite the fact that Education and White Paper 6 (2011) advocates the inclusion of learners with disability in regular schools, the findings of this study revealed that the existing public school conditions did not consider the needs of learners with disabilities, including dyslexic learners. Based on the experiences of dyslexic learners, they find comfort and recognition in special schools rather than in public schools. The findings of the study revealed that dyslexic learners felt neglected and undermined in public schools by both their peers and teachers. Paying attention to the voices of dyslexic learners, the study has revealed that the dyslexic learners prefer a special school environment to that of a public school.

### Recommendations

Based on the findings of the study, the authors recommend that the government should play an important role in creating a learning environment in public schools that is conducive for learners with dyslexia. Specifically, teachers should be equipped with sufficient skills, qualifications and competencies relevant to deal with dyslexic learners. Another important requirement is to make other learners aware that although dyslexic learners have a challenge with reading and writing, they can also be successful academically, just like any learner. This is important so that they accept and support the dyslexic learners instead of ridiculing them.
